# Space weathering effects in Bennu asteroid samples

**DOI:** 10.1038/s41561-025-01745-w

**Published:** 2025-08-22

**Authors:** L. P. Keller, M. S. Thompson, L. B. Seifert, L. E. Melendez, K. L. Thomas-Keprta, L. Le, C. J. Snead, K. C. Welten, K. Nishiizumi, M. W. Caffee, J. Masarik, H. Busemann, D. Krietsch, C. Maden, Z. Rahman, C. A. Dukes, E. A. Cloutis, Z. Gainsforth, S. A. Sandford, D. N. DellaGiustina, H. C. Connolly, D. S. Lauretta

**Affiliations:** 1 NASA Johnson Space Center, Houston, TX USA; 2 Department of Earth, Atmospheric, and Planetary Sciences, Purdue University, West Lafayette, IN USA; 3 Barrios/Jacobs, NASA Johnson Space Center, Houston, TX USA; 4 Jacobs, NASA Johnson Space Center, Houston, TX USA; 5 Space Sciences Laboratory, University of California Berkeley, Berkeley, CA USA; 6 Department of Physics and Astronomy, Purdue University, West Lafayette, IN USA; 7 Department of Nuclear Physics and Biophysics, Comenius University, Bratislava, Slovakia; 8 Institute of Geochemistry and Petrology, ETH Zurich, Switzerland; 9 Laboratory for Astrophysics and Surface Physics, Department of Materials Science and Engineering, University of Virginia, Charlottesville, VA USA; 10 Department of Geography, University of Winnipeg, Winnipeg, Manitoba Canada; 11 NASA Ames Research Center, Moffett Field, CA USA; 12 Lunar and Planetary Laboratory, University of Arizona, Tucson, AZ USA; 13 Department of Geology, Rowan University, Glassboro, NJ USA; 14 Department of Earth and Planetary Sciences, American Museum of Natural History, New York, NY USA

**Keywords:** Asteroids, comets and Kuiper belt, Mineralogy

## Abstract

The OSIRIS-REx mission deployed contact pad samplers to collect regolith from the uppermost surface of the asteroid Bennu that was exposed to the space environment. Space weathering processes, dominated by micrometeoroid impacts and solar irradiation, modify the mineralogy and chemistry of exposed surfaces to produce solar wind-amorphized layers on clays, metallic whiskers associated with high temperature melts and Fe nitride created by the reaction of indigenous N-bearing gases with space-weathered surfaces. Here, we use cosmogenic noble gases and radionuclides to suggest that the upper metre of Bennu’s regolith has been exposed to cosmic rays for 2–7 million years, consistent with remote sensing observations indicating that the asteroid’s surface is dynamic and regularly modified by mass movement. Solar energetic particle track and microcrater densities constrain the space weathering spectral changes observed in Hokioi crater to <50,000 years. These spectral changes are driven largely by the accumulation of impact melt deposits on particle surfaces, although compositional or grain size effects may also occur. Comparison of Bennu samples with those collected from the asteroids Ryugu and Itokawa suggest that micrometeoroid impacts might play a more active and rapid role in the space weathering of asteroidal surfaces than was initially suggested, particularly for carbonaceous bodies.

## Abstract (web summary)

Material from the Hokioi crater on asteroid Bennu experienced space weathering and suggests microcratering plays a more active role on carbonaceous bodies than initially thought, according to a study of OSIRIS-REx asteroid return samples.

## Main

**Extended Data Fig. 1:**
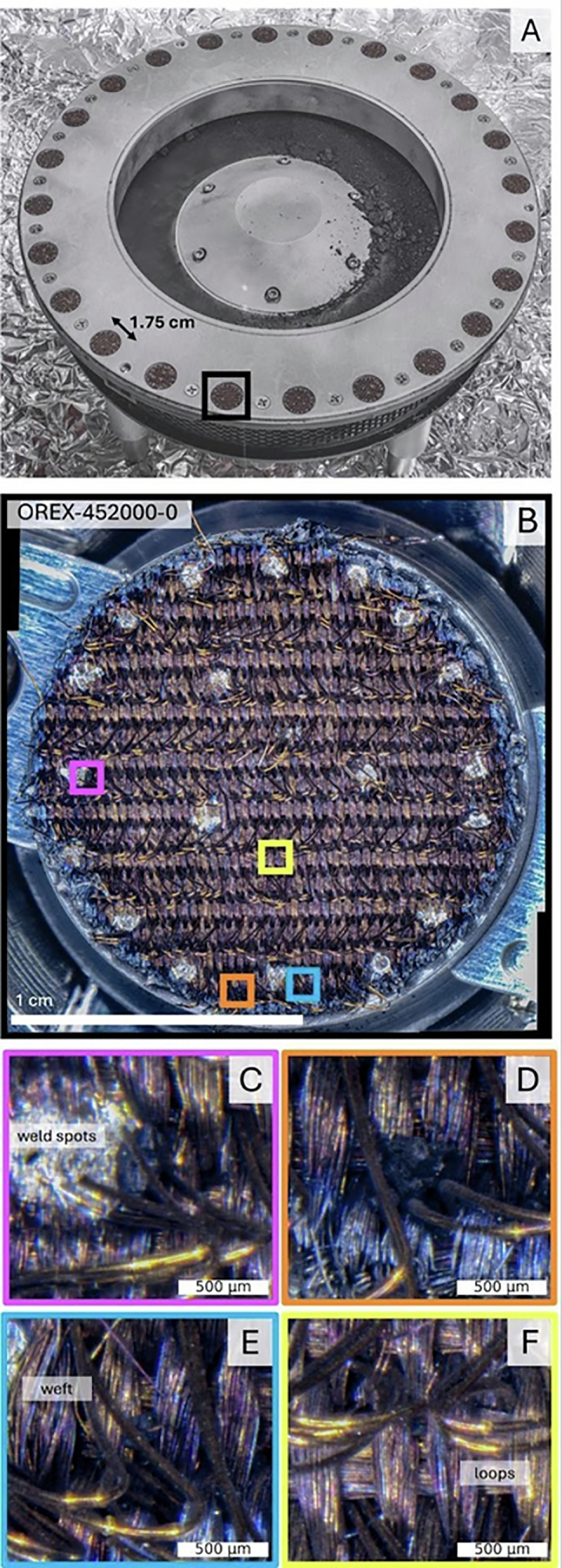
TAGSAM contact pads and associated particles. **a**, The array of contact pads arranged around the circumference of the TAGSAM base, with some of the bulk sample that can be seen in the center. **b**, Contact pad OREX-452000-0 (location on the TAGSAM indicated by the black box in panel **a**) with larger trapped particles indicated by the pink, orange, blue, and yellow boxes with higher-magnification images in panels **c-f**.

The NASA (National Aeronautics and Space Administration) Origins, Spectral Interpretation, Resource Identification, Security—Regolith Explorer (OSIRIS-REx) spacecraft returned centimetre-scale and smaller regolith particles from Hokioi crater on (101955) Bennu, a relatively rare, spectrally blue (B-type) carbonaceous asteroid^1,2^. The spacecraft’s Touch-and-Go Sample Acquisition Mechanism (TAGSAM)^3^ captured the ‘bulk’ ~122 g sample, which consists of mixed material from the surface and potentially from up to ~0.5 m in depth^4^. In addition, the TAGSAM included 24 contact pads consisting of stainless-steel Velcro, each ~17 mm in diameter and arranged symmetrically around the circumference of the TAGSAM base (Extended Data Fig. 1)^3^. Their purpose was to sample Bennu’s uppermost regolith to explore the mineralogical, chemical and spectroscopic changes resulting from exposure to space weathering.

Spacecraft data provided our first insights into the extent of space weathering effects across Bennu’s surface. Reflectance spectra showed that small craters on Bennu, especially Hokioi, are redder in the visible wavelengths than the global average; these craters were inferred to be the youngest on Bennu, with a surface exposure age estimated to be <10^5^ years (ref. ^2^). Post-sampling observations of Hokioi crater confirmed that freshly exposed materials are spectrally redder and darker in the visible wavelengths than the more space weathered, spectrally blue material exposed across most of Bennu’s surface^2,4^. However, spectra from an aliquot of the returned sample are even more red-sloped and darker than spectra collected by the spacecraft^1^, suggesting that, despite its inferred young exposure age, material at the surface of Hokoi crater has already undergone considerable space weathering.

**Fig. 1:**
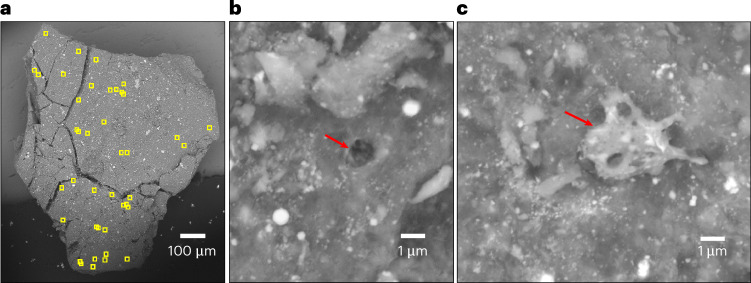
Space weathering effects in a contact pad particle. **a**, A SEM image of particle OREX-452001-0, with microcraters indicated by yellow squares. **b**,**c**, Typical microcraters (**b**) and melt deposits (**c**) on the surface of OREX-452001-0, indicated by red arrows.

Upon examination of the TAGSAM, we found that all contact pads contained adhering material, including particles captured in the Velcro loops, smaller particles in the base weave and dust particles coating much of the surface area (Extended Data Fig. 1) comprising ≪100 mg. Scanning electron microscopy (SEM) analysis shows that contact pad particles are mineralogically similar to the bulk sample^1^, including fine-grained phyllosilicates, magnetite framboids and plaquettes, Fe and Fe–Ni sulfides, carbonates, phosphates and many other minor and trace minerals^5^. SEM analyses also show that all four particles extracted thus far from contact pad OREX-452000-0, as well as tens of particles from the bulk sample, show evidence for space weathering on their surfaces, including vesicular impact melt deposits and micrometeoroid impact craters (<10 µm in diameter; Fig. 1 and Extended Data Table 1).

## Impact melt deposits

The quenched impact melt deposits (hereafter ‘melts’) are present on ~10–20% of the tens of particles analysed from the bulk sample to date and all four particles extracted from the contact pad. One contact pad particle (OREX-452001-0) exhibits ~180 individual melt deposits over ~0.6 mm^2^ (Fig. 1). The melts range in size and coverage, from splashes only a few micrometres wide to coatings covering >200 µm^2^ of the particle surface. We extracted focused ion beam (FIB) sections to investigate the microstructure and chemistry of space weathering features in the transmission electron microscope (TEM).

The bulk compositions of the melts (Supplementary Table 2) are approximately solar for most major elements (for example, Mg, Si and Fe), except S, which is depleted from the melts owing to its volatility, resulting in the formation of Fe metal grains in some melts. The melts also contain abundant nano- and micro-phase inclusions of Fe–Ni–S phases and can also include larger immiscible Fe–Ni–S melts, either as discrete deposits or as irregular swirls (schlieren) in the host silicate melts.

In addition to micro- and nano-phase inclusions, there are widespread vesicles throughout the melts (~500 nm in diameter) and also concentrated at the melt interface with the phyllosilicate-rich matrix (~50 nm in diameter). The large vesicles resemble those in melts produced by infra-red laser irradiation of hydrated carbonaceous chondrites—where the vesicles form from evolved volatiles (mainly H_2_O)^6^. This H_2_O probably contributes to the broadening of the 2.7-µm water feature observed in the spacecraft spectra^7^. The smaller vesicles resemble those observed in solar wind-irradiated surfaces of particles returned from Itokawa^8^ and laboratory experiments^9,10^.

**Fig. 2:**
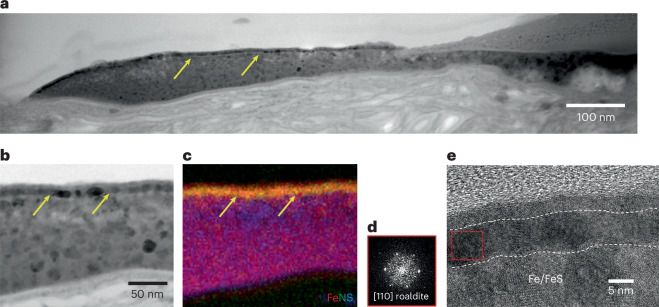
An iron nitride layer on the surface of a Fe–Ni–S melt. **a**, A TEM image of a FIB section of particle OREX-501017-101, with the iron nitride layer indicated by yellow arrows. **b**, A higher-magnification image of the polycrystalline layer. **c**, The Fe–N–S (RGB) composite chemical map of the area shown in **b**. **d**,**e**, A high-resolution TEM image (**e**) showing that the Fe nitride is crystalline and a fast Fourier transform pattern (**d**) from the inset red box, consistent with the mineral roaldite.

In contrast to the silicate melts described above, the Fe–Ni–S melt deposits are crystalline and dominated by fine-grained mixtures of pyrrhotite, pentlandite, FeNi metal and minor magnetite. On one such melt deposit (OREX-501017-101), we observed a polycrystalline ~10-nm Fe nitride layer (Fig. 2) that consists of an intergrowth of siderazot (Fe_3_N_1.3_) and roaldite (Fe_4_N). The Fe nitride minerals probably formed through a gas-phase reaction of the Fe metal layer with a N-bearing volatile phase, most probably indigenous ammonia^11^. The discovery of nitrides in Ryugu^12^ and Bennu samples suggests an unexpected but possibly common process involving interactions between ammonia and the space-weathered regolith particles on carbonaceous asteroids.

**Fig. 3:**
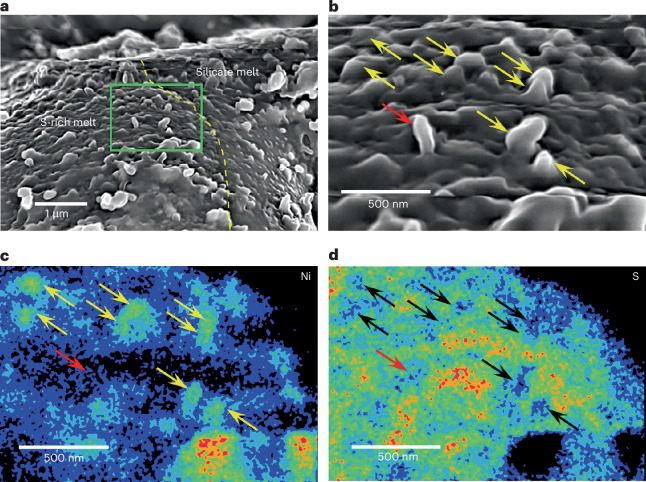
FeNi metal whiskers that have developed on sulfide-rich melt deposits. **a**, A SEM image of particle OREX-803164-0 containing a sulfide-rich melt droplet (labelled ‘S-rich melt’). The dashed yellow line indicates the boundary between the sulfide melt and the silicate melt. The green box indicates a magnified area shown in the subsequent panels. The red and yellow arrows indicate a Ni-poor whisker and a Ni-rich whisker, respectively. **b**, Yellow arrows point toward Ni-rich whiskers, more of which can be seen in this magnified view, and the red arrow points towards the Ni-poor whisker. **c**,**d**, Corresponding Ni (**c**) and S (**d**) maps showing that the whiskers are Ni-rich and S-depleted. Arrows pointing towards Ni-rich whiskers in **d** are coloured black for clarity.

A sulfide melt on particle OREX-803164-100 lacks an Fe nitride layer; however, on that deposit, we observe the formation of multiple FeNi-rich metal whiskers protruding up to ~100 nm from the surface (Fig. 3), like those observed on sulfides in samples from Itokawa^13^ and the Moon^14^ (see ‘Impact melt deposits’ section in the Supplementary Data).

## Solar wind irradiation

Evidence for solar wind irradiation was identified in particles that exhibit other characteristics consistent with surface exposure on Bennu (for example, microcraters and melts). In addition, we examined areas immediately underlying melts superimposed on previously solar wind-exposed surfaces.

**Extended Data Fig. 2:**
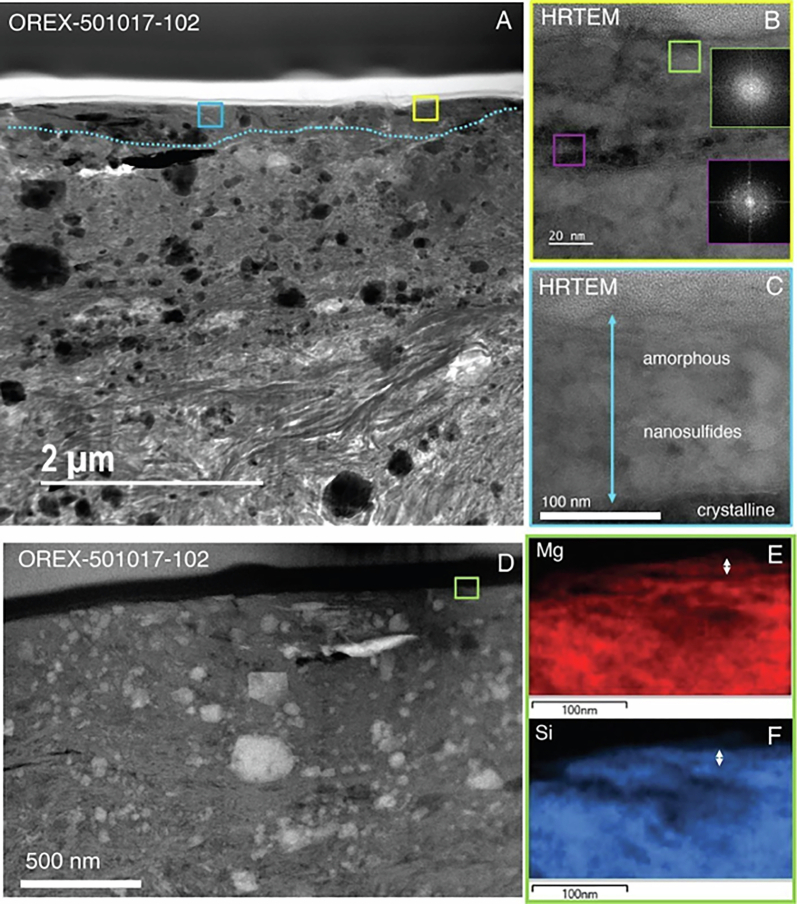
TEM evidence for solar wind irradiation. **a**, Bright-field scanning transmission electron microscope (BF STEM) image of particle OREX-501017-102 showing a porous phyllosilicate matrix with a contact surface layer outlined by the blue dashed line. The yellow and blue boxes indicate the areas shown in panels b and c, respectively. **b**, High-resolution TEM (HRTEM) image showing the amorphous texture of the rim (green FFT inset) which transitions to nanocrystalline (purple FFT inset). **c**, HRTEM image showing an area of the rim that is completely amorphous and >100 nm thick. **d**, HAADF image of particle OREX-501017-102. Bright phases are sulfides and magnetite. The green box shows the location of EDS maps (insets) showing chemical segregation in the solar wind–irradiated rim, with an enrichment in (**e**) Mg and a depletion in (**f**) Si in the uppermost 10 nm of the rim, with the respective layers shown by the white arrows.

On particle OREX-501017-102, we observe a less porous, compact surface layer, ranging in thickness from 60 to 150 nm, consistent with the expected implantation depths for solar wind H^+^ and He^+^ in silicate materials estimated by Monte Carlo atomic collision modelling^9,15^. This compact layer exhibits heterogeneous crystallinity, with a predominantly amorphous surface transitioning to poorly crystalline, and ultimately long-range crystalline order at the base (Extended Data Fig. 2). The compact surface layer is also chemically processed, with a 10-nm-thick rim enriched in Mg and depleted in Si, overlying a similar layer depleted in Mg and enriched in Si. This chemical signature is commonly observed in other solar wind-irradiated returned samples and analogues^9,10,16^ (see ‘Solar wind irradiation’ section in the Supplementary Data).

**Fig. 4:**
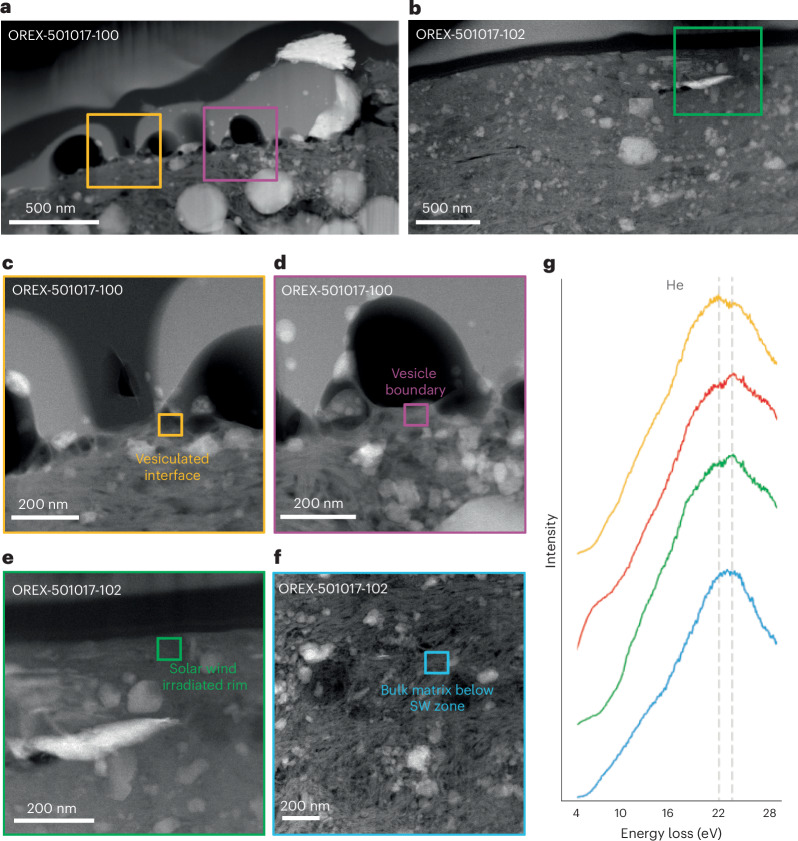
TEM data showing evidence for solar wind irradiation. **a**,**b**, High-angle annular dark-field (HAADF) images of the OREX-501017-100 (**a**) and OREX-501017-102 (**b**) samples analysed with EELS, with measurement locations identified by coloured boxes. **c**,**d**, Interface between melt deposits and the underlying matrix in particle OREX-501017-100 from different regions where the EELS spectra were obtained, and shown in **g**. **e**, A HAADF image of the solar wind-irradiated rim in particle OREX-501017-102. **f**, A HAADF image of the matrix, below the implantation depth for solar wind, in particle OREX-501017-102 (not shown in **a** and **b**). SW, solar wind. **g**, EELS spectra (arbitrarily offset in intensity only for clarity) showing the presence of He, with spectrum colours corresponding to the boxes in **c**–**f**. The peak positions for the He K-edge shifts to higher energies (between 22 and 23 eV, as indicated by the dashed grey lines) with higher concentration detected^36^.

We directly measured solar wind-derived He in particles that showed evidence for surface exposure (OREX-501017-102 and OREX-501017-100). Electron energy loss spectroscopy (EELS) measurements revealed heterogeneously distributed He in the compact surface layer of particle OREX-501017-102 (Fig. 4). We also identified He in OREX-501017-100 in small vesicles concentrated at the melt–matrix interface. These observations support the inferred relationship between interface vesicles and the implantation of solar wind gases.

**Fig. 5:**
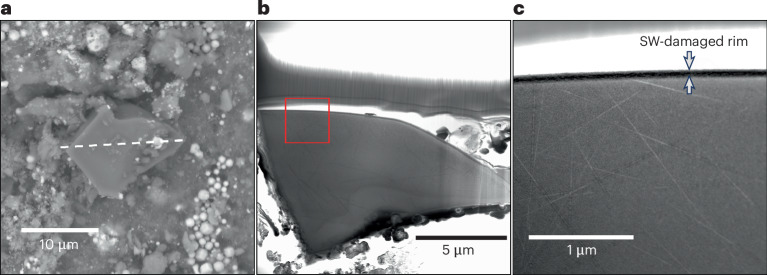
A track-rich forsterite grain on the surface of particle OREX-452001-0. **a**, A SEM image with the forsterite grain in the centre and a dashed line indicating the orientation of the FIB section. **b**, A TEM image of the FIB cross section of the forsterite. **c**, An expanded TEM image from the red outlined area in **b**, showing the solar wind damage on the rim and SEP tracks (linear features) in the grain.

Olivine and pyroxene grains exposed to the space environment are rare in Bennu samples^1,5^. We extracted a FIB section from a forsterite grain on the surface of a contact pad particle (OREX-452001-0) and observed a nanocrystalline solar wind-damaged rim ~100 nm wide and solar energetic particle (SEP) tracks with a density of ~1.4 × 10^9^ cm^−2^ (Fig. 5). The rim microstructure is similar to solar wind-damaged rims in olivine grains from Itokawa and the Moon with similar SEP track densities^17^.

## Micrometeoroid impact craters

**Fig. 6:**
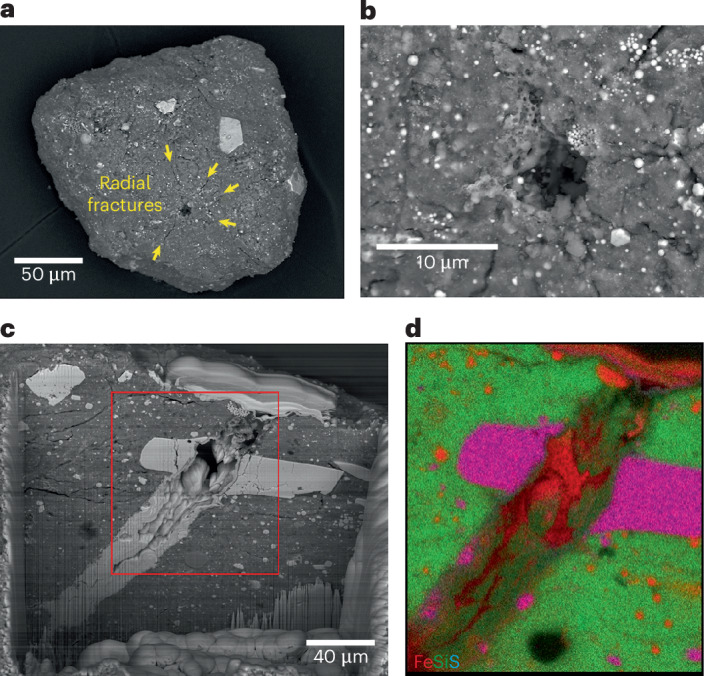
Micrometeoroid impact crater in particle OREX-803109-0. **a**, A SEM image of the particle showing the crater (dark area) and the radial fractures surrounding it (yellow arrows). **b**, The crater entrance is lined with vesiculated melt. **c**, The crater as exposed in the FIB trench before extracting a section for TEM analysis. The red box indicates the area shown in **d**. **d**, The Fe–Si–S (RGB) composite chemical map of the crater path through matrix material, including a platy pyrrhotite grain (magenta).

We used SEM imaging to identify micrometeoroid impact craters on the basis of their circular morphology, raised rims and the presence of vesicular melt deposits lining the crater floors (Fig. 1b). Micrometre-sized impact craters are commonly observed on contact pad particles and on some particles from the bulk sample. On the surface of contact pad particle OREX-452001-0, we observe ~40 microcraters with diameter typically 1–2 μm. In contrast, the largest crater we have identified is in a particle from bulk sample OREX-803109-0 and is ~10 μm in diameter with radial fractures extending outwards (Fig. 6).

## Exposure ages

We use solar energetic particle tracks and microcrater densities to constrain recent direct exposure at Bennu’s surface (less than millimetre depths). At greater depths (upper few metres), we use the production of the radioactive cosmogenic nuclides (hereafter ‘radionuclides’) ^10^Be and ^26^Al as well as the stable noble gas nuclide ^21^Ne by exposure to galactic cosmic rays. Whereas cosmogenic noble gases record the total cosmic ray exposure (CRE) history of the samples, radionuclides record the CRE history of the past 5–10 Myr.

Solar energetic particles leave tracks of ionization damage in anhydrous silicate crystals, and their density can be used as a chronometer for exposure in the uppermost millimetres of the regolith^18^. The SEP track density in the forsterite exposed on OREX-452001-0 (~1.4 × 10^9^ cm^−2^) is consistent with an exposure age of ~45,000 years (Methods).

Microcrater densities also constrain the surface exposure age of particles. While contact pad particle OREX-452001-0 has the highest microcrater density, this estimate represents a minimum density for this particle because we did not count micrometre-sized pits without melt as microcraters. We use this density to estimate the exposure age by comparison with the cumulative microcrater production rate observed for lunar rocks and soils^19^, giving a range in exposure age of ~20,000–85,000 years (Methods).

Bennu particles (Methods), such as those returned from Ryugu^20^, contain abundant primordially trapped noble gas components that mostly overprint the much less abundant cosmogenic noble gases^21^. Some particles that were exposed at Bennu’s surface also contain abundant trapped solar wind, evident in He and Ne. Because the trapped components are so dominant, any resolvable excesses of cosmogenic ^21^Ne are minimal if present at all. Otherwise, upper limits were determined (see data in ref. ^21^). Where resolvable, the concentration of cosmogenic ^21^Ne in the Bennu particles (2.8 and 4.2 × 10^−9^ cm^3^ STP g^−1^, where STP is standard temperature and pressure) suggests a CRE age of 2–7 Myr, depending mostly on the depth where the exposure to galactic cosmic rays occurred.

We measured the concentrations of the cosmogenic radionuclides ^10^Be (half-life of 1.36 Myr (ref. ^22^)), ^26^Al (half-life of 0.705 Myr (ref. ^23^)) and ^36^Cl (half-life of 0.301 Myr (ref. ^24^)) in aggregate sample OREX-803047-0. (Supplementary Table 3). The cosmogenic ^10^Be and ^26^Al concentrations correspond to irradiation depths of 170–180 and 100–110 g cm^−2^, respectively^25^. Since the collected sample is probably a mixture of materials from the surface and deeper in the crater^26^, scenarios where individual particles were exposed at different depths and for different durations may explain the cosmogenic nuclide record of the sample. Regardless of the different histories of individual particles, the cosmogenic radionuclide results indicate a minimum CRE age of >3 Myr for the sample, which is consistent with the ages >2 Myr derived from cosmogenic ^21^Ne here. Nevertheless, some caution is needed as noble gas measurements were performed on different samples than those used for radionuclide measurements.

If the 2–7 Myr CRE age of the samples that we examined is representative of Bennu’s average surface, then the exposure time of the top few metres of regolith is less than the asteroid’s oldest estimated age (~1 Gyr) by more than two orders of magnitude^27^. This indicates that the uppermost few metres of Bennu are frequently replenished with material from depths >5–10 m, probably by a combination of impact gardening and downslope mass movements. This is consistent with spacecraft observations indicating a dynamic surface, including particle ejection events (for example, ref. ^28^), mass movement within the past few hundreds of thousands of years^27,29^ and a crater size frequency distribution indicating that the upper ~10 m of Bennu is resurfaced on a timescale of 10–65 Myr (ref. ^30^). Finally, our initial results for Bennu materials indicate a CRE age of approximately 5 million years, similar to the age determined for Ryugu^20^, suggesting that these two asteroids have experienced comparable surface evolution histories.

## Connecting returned samples to spacecraft observations

With the analysis of samples from Bennu, we can compare weathering timescales from a remote versus laboratory perspective, while also linking spectral characteristics to their microstructural and chemical sources. The younger SEP exposure age we find from a particle collected at the surface (~10^4^ years) suggests that space weathering changes occurred on a more rapid timescale than was estimated from spacecraft data (10^5^ years).

Spacecraft measurements also suggest that surface material on Bennu becomes brighter and bluer with continued exposure to the space environment, in contrast to lunar and ordinary chondritic materials^2,3,31^. Compositionally, Bennu samples contain hydrated amorphous Mg,Na phosphate^1,32^; a similar phase in Ryugu samples displays a blue slope across visible wavelengths^33^, suggesting that such material may be contributing to Bennu and Ryugu reflectance spectra. In laboratory studies with analogue materials, the bluing trend has been attributed to a variety of optically opaque minerals, including carbonaceous materials, sulfides or magnetite^6,34^. The melt deposits on the surfaces of Bennu particles contain abundant nano-phase and micro-phase inclusions of FeNi metal and FeNi sulfides. Spectral modelling of troilite (FeS) inclusions in silicate hosts indicate that particles >40 nm in diameter cause bluing of reflectance spectra across visible–near-infra-red wavelengths^34^. This link between nano-phase sulfides, which we observe in the samples, and increasingly blue spectral characteristics offers a fresh paradigm for space weathering on airless carbonaceous bodies where nano- and microscale sulfide inclusions influence the evolution of spectral properties observed through remote sensing, rather than the nano-phase Fe metal. These observations provide context for space weathering processes across the Solar System and may have implications for rocky bodies enriched in sulfur, such as Mercury.

## An evolving model for space weathering in the inner Solar System

Space weathering is a ubiquitous process affecting airless bodies across the Solar System, and understanding the relative contributions of its constituent processes and their timescales is critical for interpreting the mineralogy and evolution of these planetary surfaces. The canonical model for space weathering constructed from lunar observations suggested that solar wind modifies airless surfaces on short timescales (10^4^–10^6^ years), whereas impacts operate on longer timescales (10^8^–10^9^ years)^35,36^. This was reinforced by observations from Itokawa samples, where solar wind appeared to be the dominant space weathering agent, acting to alter spectral characteristics more efficiently and on shorter timescales than impacts^8,36^.

However, we can now compare space weathering characteristics in returned samples across all our asteroidal collections (Itokawa, Ryugu and Bennu). Melt deposits occur in <0.5% of Itokawa samples^37^, 2% of Ryugu particles^16^ and 20% of Bennu particles (although analyses of additional material may improve these statistics). In contrast, Ryugu samples have the lowest SEP track density ages (6,000 years), compared with those from Bennu (45,000 years) and Itokawa (50,000 years)^8,16^. Together, these results suggest that micrometeoroid impacts play a more important role in the space weathering of asteroidal surfaces than was suggested from early observations of asteroidal returned samples.

Comparing space weathering characteristics between only Ryugu and Bennu samples reveals a common suite of products, including vesiculated melts with nano- and micro-phase FeNi sulfide inclusions, solar wind-damaged rims, microcraters and the unusual metal whiskers and nitride layers described above^12,13,16,17^. This is unsurprising given their mineralogical and compositional similarity and their exposure to the near-Earth space environment. However, the observed SEP track and microcrater densities indicate that Bennu samples have an order-of-magnitude longer surface exposure than Ryugu samples. In addition, there are apparent differences in the spectral slopes, colours and strength of the 2.7-µm hydration feature between Ryugu and Bennu in spacecraft data^31^.

The spectral slope differences between Ryugu and Bennu^31^ were attributed to different space weathering trends operating on the bodies, but the asteroids’ similar chemical and microstructural characteristics and the age relationship we infer from the samples indicates that the space weathering trend is a single path with spectral slopes evolving from red to blue over time. Ryugu sits at an earlier stage of this pathway, and we predict that it will eventually develop a bluer, Bennu-like spectral slope. Other factors such as particle size, minor mineralogical differences or rock surface textures may also contribute to differences between the asteroids.

The space weathering characteristics of Bennu and Ryugu revealed by remote sensing and sample analysis suggest that space weathering is fundamentally different for primitive carbonaceous bodies compared with the Moon and S-complex asteroids in four ways:

1. Micrometeoroid impacts are more efficiently driving rapid spectral changes relative to solar wind effects on hydrated carbonaceous asteroids, which also contrasts with existing models for inner Solar System weathering timescales (see, for example, ref. ^36^).
2. Observations from Bennu samples have demonstrated that impacts not only alter the surfaces of C-complex asteroids more substantially but do so on a far shorter timescale than hypothesized on the basis of lunar surfaces (10^4^ years compared with >10^8^ years).
3. The effects of micrometeoroid impacts are known to be magnified by the low mechanical strength^38^, higher volatile content and more efficient melting of carbonaceous surfaces versus their anhydrous lunar and asteroidal counterparts. These properties result in the wide dispersion of melt ejecta by shocked volatiles^39^.
4. Spectral slopes are driven towards bluing with continued exposure, rather than reddening, a reversal of lunar-style space weathering spectral trends. Differences in surface exposure timescales between Ryugu (shorter) and Bennu (longer) support the hypothesis that carbonaceous asteroids experience a pathway of weathering driven by early reddening, followed by eventual bluing^2^. Bennu and Ryugu are snapshots of asteroids at two different stages of this pathway, with Ryugu exhibiting a redder spectral slope, lower crater density and younger surface exposure ages, and Bennu showing a bluer slope, higher crater densities and longer surface exposure ages. These observations provide another metric by which relative surface ages could be determined for carbonaceous asteroids in the main belt.

Recent crater distribution and retention age analyses^40^ confirm that rubble-pile asteroids can experience exceptionally rapid regolith mixing and resurfacing, with turnover timescales on Ryugu of 10^3^–10^5^ years. This rapid cycling suppresses long-term spectral bluing and promotes the exposure of fresh material, especially on Ryugu, where younger surface ages result in a redder spectral slope. Bennu, having experienced fewer resurfacing events, has transitioned further along the spectral weathering pathway towards bluer slopes. These processes, driven by impact gardening and surface flows, appear to dominate space weathering on carbonaceous asteroids, fundamentally distinguishing them from those on the Moon and S-type asteroids.

## Methods

Evidence for space weathering on particle surfaces (for example, vesicular melt deposits with embedded nano-particles) was identified using SEM of particles extracted from contact pad OREX-452000-0 and from aggregate samples OREX-501017-0, OREX-803081-0 and OREX-803164-0 (where ‘aggregate’ signifies a sample consisting of unsorted particles). We used a JEOL 7900F scanning electron microscope at NASA Johnson Space Center (JSC) operated in low vacuum mode to image the contact pad particles without a conductive coating. For other aggregate samples, we used a JEOL 7600F field-emission scanning electron microscope also at JSC. To prepare the samples by FIB milling, we used an FEI Quanta3D at JSC and the FEI Helios Nanolab 660 G^3^ in the University of Arizona’s Kuiper-Arizona Laboratory for Astromaterials Analysis (K-ALFAA). TEM analysis of the microstructural and chemical characteristics of space weathering was performed using the JEOL 2500SE TEM at JSC, the aberration-corrected Hitachi HF5000 in K-ALFAA and the aberration-corrected, monochromated Thermo Themis Z at Purdue. Each TEM is equipped with a silicon drift detector for energy-dispersive X-ray spectroscopy and a Gatan EELS for chemical analyses.

The complete noble gas data set, obtained at ETH Zurich with a custom-built sector-field mass spectrometer (all isotopes of He–Xe), is presented in a companion paper^21^. The cosmogenic ^21^Ne concentrations used here were derived by two-component decomposition of the measured Ne composition between the trapped end-member and cosmogenic Ne typical for material of CI chemical composition. Samples were transported to ETH from the NHM London in N_2_ atmosphere. They were subsequently handled in a N_2_-filled glove box, weighed using a Mettler Toledo UMX2 ultra-microbalance in hermetically sealed micro-weighing containers and loaded into an infra-red laser cell without any exposure to air. Three particles (OREX-800032-102, OREX-800032-103 and OREX-800032-104) were placed individually into the laser chamber and pumped down to 10^−10^ mbar. Each particle was melted completely in two steps using a Nd:YAG laser working at 1,064 nm wavelength. Gases released upon exposure to the infra-red laser were purified, cryogenically separated into He–Ne, Ar and Kr–Xe and analysed according to standard procedures^41^.

We received ~12 mg of an aggregate sample OREX-803014-0 of loose, unsorted material <1 mm in size for analysis of cosmogenic radionuclides by accelerator mass spectrometry (AMS). The sample was split into 5-mm-sized particles and a bulk sample, OREX-803047-0, of 9.85 mg. We dissolved the bulk sample along with Be and Cl carriers in a HF/HNO_3_ mixture. After dissolution, we separated Cl as AgCl for analysis of cosmogenic ^36^Cl by AMS. A small aliquot of the dissolved sample was split for chemical analysis by inductively coupled plasma optical emission spectrometry. The chemical composition of the sample (Supplementary Table 3) is consistent with that of a similar aggregate sample, OREX-803015-0, measured previously by others^1^. We then added 1.5 mg of Al carrier and separated Be and Al from the remaining solution for analysis of cosmogenic ^10^Be and ^26^Al by AMS. The AMS measurements of the ^10^Be/Be, ^26^Al/Al and ^36^Cl/Cl ratios were performed at the Purdue Rare Isotope Measurement Laboratory^42^. The measured ratios were normalized to those of well-known AMS standards^22–24^. From the normalized ratios, the amount of Be, Al and Cl carrier, and the sample mass, we calculated the ^10^Be, ^26^Al and ^36^Cl concentrations of the sample. Concentrations (in atoms per gram) wre then converted to disintegrations per minute per kg and are reported in Supplementary Table 3. The measured concentrations of ^10^Be and ^26^Al are compared with production rates for 2π irradiation from model calculations^25^, adopting the bulk composition of Bennu aggregate samples^1^. If the measured concentrations represent saturation values during steady-state exposure conditions, they provide a direct measure of the irradiation depth of the sample.

We determined a SEP track exposure age from the track measurements in OREX-452001-0 by using the 1 AU track production rate calibration from ref. ^18^ and an *r*^−1.7^ model^43^ for the heliocentric decay of the SEP flux to account for the parts of Bennu’s elliptical orbit beyond 1 AU, where *r* is the heliocentric radius. The measurements were made on a FIB section prepared using the JSC FIB and images obtained using the JEOL 2500SE STEM at JSC.

The microcrater exposure age for OREX-4520001-0 was determined by measuring the number of definite craters in the measured area (~40 craters in 0.6 mm^2^ of surface area) and using the crater production rates from ref. ^19^ of ~0.3 craters per square centimetre per year for 2-μm-diameter craters and 0.08 craters per square centimetre per year for 1-μm craters (for a 2π exposure), giving exposure ages ranging between ~20,000 and 85,000 years. However, this estimate represents the minimum crater density for this particle because we did not count micrometre-sized pits or other circular features without melt as microcraters.

## Online content

Any methods, additional references, Nature Portfolio reporting summaries, source data, extended data, supplementary information, acknowledgements, peer review information; details of author contributions and competing interests; and statements of data and code availability are available at 10.1038/s41561-025-01745-w.

## Supplementary information

Supplementary Information Supplementary Discussion and Tables 1–3.

## Figures and Tables

**Extended Data Table 1:**
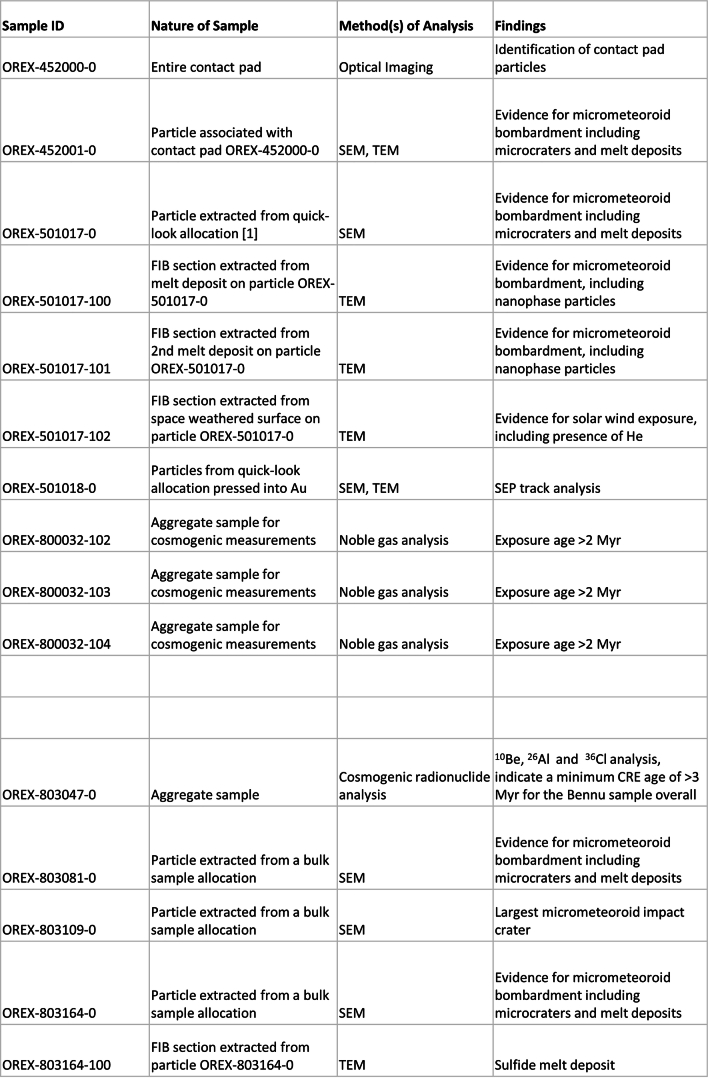
Sample descriptions, analytical techniques, and outcomes

## Data Availability

Instrument data supporting the experimental results from the samples analyzed in this study will be available via Astromat (astromat.org) at the DOIs listed in Supplementary Table 1 and below and are available within the manuscript or the Supplementary Information.
