# Supplementary material for: Space weathering effects in Bennu asteroid samples
Source: Nat Geosci. 2025 Aug 22;18(9):825–31. doi: 10.1038/s41561-025-01745-w (PMC12422962; doi:10.1038/s41561-025-01745-w)
Supplement: Supplementary file 1 — Supplementary Discussion and Tables 1–3. [file 41561_2025_1745_MOESM1_ESM.pdf]

nature geoscience

Supplementary information https://doi.org/10.1038/s41561-025-01745-w

Space weathering effects in Bennu asteroid samples

In the format provided by the authors and unedited

SUPPLEMENTAL TEXT: Nature Geoscience Manuscript NGS-2024-11-03082a

Impact Melt Deposits

We analyzed melt deposits in focused ion beam (FIB) sections using TEM to characterize their microstructure and chemistry. The melts typically contain nano- and micro-phase inclusions of mixed FeNi metal and FeNi sulfides. These inclusions range in size from a few nanometers up to a micrometer. Many have eutectic-melt compositions with FeNi-metal cores surrounded by FeS rims, while a few are hollow. The larger sulfide inclusions within the silicate glass are flattened parallel to the particle surface. We have not observed any glass-welded (e.g., agglutinate-like) particles like those commonly observed in space-weathered lunar materials [1], although rare impact spherules are observed attached to grain surfaces. As described in the main text, we observe the formation of Fe-nitrides on one of the FeNiS melt deposits including the minerals roaldite and siderazot. Siderazot is a rare mineral that occurs terrestrially in volcanic fumarole deposits [2]. The sulfide melt was irradiated by the solar wind, resulting in preferential loss of sulfur via sputtering, which produced a thin surface layer of Fe-metal, similar to irradiation effects observed in sulfide grains from Itokawa [3,4]. We believe these minerals form by reaction of Fe metal with indigenous ammonia. In contrast, Fe-nitride layers on magnetite grains returned from the carbonaceous asteroid Ryugu by the Hayabusa2 mission are proposed to originate from the reaction with exogenous N-rich ices from the outer Solar System [5].

We analyzed an additional FeNiS melt deposit and discovered numerous FeNi metal whiskers growing from the melt surface (Fig. 3). These whiskers have been attributed to solar wind irradiation processes [6,7], wherein implanted solar wind hydrogen reacts with S in the target to form H_2S gas, reduces constituent Fe to metal, and eventually exsolves from the sample. This process may result in the formation of filamentous whisker structures that serve as microstructural pathways or conduits for efficient gas diffusion [6]. Here, however, the presence of Ni-rich whiskers on melt deposit surfaces suggests a direct relationship between their formation and impact processes. Compressive stresses, which can develop in particle surfaces because of micrometeoroid impacts, have led to whisker growth in a variety of substrates [8-11]. These observations suggest that micrometeoroid impacts produce unique microstructural characteristics in sulfide minerals and may reveal a hidden record of impact processes in returned samples from other small bodies, previously attributed to solar irradiation.

Micrometeoroid Impact Craters

For the microcrater shown in Fig. 6, we prepared a FIB cross section through the upper part of this crater. The crater rim contains abundant vesiculated melt with embedded nanophase sulfide-rich grains distributed throughout. In the FIB section, the crater diameter changes little with the depth, which exceeds 70 µm from the surface of the particle. At a depth of ~10 µm below the particle surface, the impactor completely penetrated a 10 µm thick pyrrhotite plate and continued its trajectory, uninterrupted, deeper into the phyllosilicate matrix of the particle. Melt deposits line the impact channel and are composed of mixed silicate-sulfide melts with abundant vesicles and embedded sulfide-rich nanoparticles. We hypothesize that the impactor was approximately micrometer-sized, mechanically strong, and traveling at high velocity (>5 km/s) to penetrate so deeply and remain intact. This hypothesis is supported by experiments with refractory, high-strength projectiles impacting low-density, porous target materials, where target melting along the impact channel allows deeper penetration of the impactor and the formation of long cylindrical tracks [12].

Solar Wind Irradiation

Compact surface layers attributed to solar wind irradiation have been identified in Bennu samples. In contrast to underlying phyllosilicates, which typically exhibit a fluffy and/or porous texture, the compact surface layers appear denser, with little porosity. These layers are also chemically processed with surface enrichments of Mg and depletions of Si, as described in the main text. The chemically heterogeneous layer is attributed to a number of ion-processing effects, including recoil implantation, sputtering, and/or radiation-enhanced diffusion or segregation. The silicate melt deposit in OREX-501017-101 also exhibits this same Mg-depleted, Si-rich layer at the surface.

Despite observations of abundant vesicles in impact melts and at melt-sample interfaces, we have not observed the surface blistering and vesiculation textures that are commonly observed on particles from Itokawa [13] and in experimentally irradiated analogs. As surface vesiculation is linked to both irradiation flux and fluence [14,15], its absence is consistent with the hypothesis that Bennu particles have short surface exposure histories compared to their counterparts from Itokawa and the Moon.

Exposure Ages

A group of forsterite grain in particle (OREX-501018-0) from the bulk sample lacked SEP tracks, indicating a short SEP exposure age of <400 years, or more likely, that the grains were buried in the Bennu regolith beyond the penetration depth of SEPs (~<5 mm) for most of the particle's residence time on Bennu.

The lower depth estimate derived from ^{10}Be is most easily explained by a short CRE age, which would imply that ^{10}Be was not saturated. Correcting ^{10}Be for a CRE age of approximately 3 Myr yields an irradiation depth of 100 ± 20 g/cm^2, consistent with the irradiation depth derived from ^{26}Al This depth is consistent with the relatively wide range of ~30-200 g/cm^2 indicated by the high concentration of ^{36}Cl (~91 dpm/kg) which is dominated by neutron capture on ^{35}Cl. If this irradiation took place before the formation of Hokioi crater, i.e., at a depth of ~3 m [16], it implies that the density of the regolith is <0.5 g/cm^3 — lower than the bulk density of Bennu, and lower than estimates of 0.5-0.7 g/cm^3 derived from the spacecraft's contact with Bennu's surface during sampling [17].

A Model for Space Weathering in the Inner Solar System

We have noted a difference in the space weathering extent in Ryugu versus Bennu samples. Here we consider the differences in the hydration features. For Ryugu samples, the abundance of melt deposits likely resulted in the OH/H_2O loss (dehydration) inferred from spacecraft measurements of the 2.7 µm hydration feature [18], which is ~50% of the intensity of that feature in the returned samples [19]. However, the hydration feature in Bennu samples is strong [20,21], and while the hydration bands observed by the OSIRIS-REx spacecraft are broader than in the laboratory measurements, the band depth is similar, apparently consistent with limited H_2O loss from surface materials despite the longer exposure timescale compared to Ryugu. These observations indicate that space weathering on Bennu was sufficient to alter the optical properties of the regolith samples on a short timescale (~10^4 years), but not to attenuate major spectroscopic features (e.g., OH/H_2O) in remote sensing data, as was apparently the case on Ryugu.

The origin of the differences in the hydration feature band depth between Ryugu and Bennu remains unexplained. One possibility is that the surface of Bennu could have been rehydrated over its longer exposure timescales via irradiation from the solar wind. The implantation of H^+ ions into oxygen-bearing minerals in the regolith has been shown to produce water (i.e., OH^- and H_2O) on the surfaces of airless bodies [22]. This mechanism results in the slow and steady production of water, which could accumulate in the regolith over time, in the absence of any outgassing of dehydration events. This process may require >10^4 years to produce water at a rate which outpaces the more stochastic impact events driving surface dehydration. We know from observations of Bennu samples that some melt deposits on grain surfaces exhibit evidence for solar wind irradiation, subsequent to the melt emplacement. These complex rims may provide microstructural evidence of the competing processes affecting surface water content. Analysis of lunar samples from the Chang'e-5 mission revealed measurable absorption bands attributed to water sourced from the solar wind in grains exposed on the order of 10^3 years

[23]. These results suggest that a 10^3–10^4 year exposure timescale, which straddles the boundary between Ryugu and Bennu, may be the turning point at which dehydration via impacts begins to be balanced by rehydration via solar wind. Such a mechanism would also explain why airless surfaces do not experience runaway dehydration.

References

[1] Denevi, B. W., Noble, S. K., Christoffersen, R., Thompson, M. S., Glotch, T. D., Blewett, D. T., Garrick-Bethell, I., Gillis-Davis, J. J., Greenhagen, B. T., Hendrix, A. R., Hurley, D. M., Keller, L. P., Kramer, G. Y., & Trang, D. (2023) Space weathering at the Moon. In New Views of the Moon - 2 (ed. C. Neal et al.). Reviews in Mineralogy & Geochemistry, 89, 611-650. doi.org/10.2138/rmg.2023.89.14

[2] Bette, S., Theye, T., Bernhardt, H.-J., Clark, W.P. & Niewa, R. Confirmation of siderazot, $Fe_3N_{1.33}$, the only terrestrial nitride mineral. Minerals: 11(3): 290. doi.org/10.3390/min11030290 (2021).

[3] Keller, L. P. & Berger, E. L. A transmission electron microscope study of Itokawa regolith grains, Earth Planets Space, 66, 71-80 (2014).

[4] Chaves, L.C. and Thompson, M.S. Space weathering signatures in sulfide and silicate minerals from asteroid Itokawa. Earth, Planets and Space, 74, 124 (2022).

[5] Matsumoto, T. et al. Influx of nitrogen-rich material from the outer Solar System indicated by iron nitride in Ryugu samples, Nature Astronomy, 8, 207-215 (2024).

[6] Matsumoto, T., Harries, D., Langenhorst, F., Miyake, A. & Noguchi, T. Iron whiskers on asteroid Itokawa indicate sulfide destruction by space weathering. Nature communications, 11(1), 1117 (2020).

[7] Matsumoto, T., Noguchi, T., Tobimatsu, Y., Harries, D., Langenhorst, F., Miyake, A. & Hidaka, H. Space weathering of iron sulfides in the lunar surface environment. Geochimica et Cosmochimica Acta, 299, 69-84 (2021).

[8] Tu, K. N. Irreversible processes of spontaneous whisker growth in bimetallic Cu-Sn thin-film reactions. Physical Review B, 49, 2030-2034(1994).

[9] Dudek, M. A.& Chawla, N. Mechanisms for Sn whisker growth in rare earth-containing Pb-free solders. Acta Materialia 57, 4588–4599 (2009).

[10] Williams, J. J., Chapman, N. C. & Chawla, N. Mechanisms of Sn hillock growth in vacuum by in situ nanoindentation in a scanning electron microscope (SEM). Journal of Electronic Materials, 42, 224-229 (2013).

[11] Guo, L., Zhong, S., Bao, Q., Gao, J. & Guo, Z. Nucleation and growth of iron whiskers during gaseous reduction of hematite iron ore fines. Metals 9, 750; doi:10.3390/met9070750 (2019).

[12] Hörz, F., Cintala, M.J., See, T.H. & Nakamura-Messenger, K. Penetration tracks in aerogel produced by Al2O3 spheres. Meteoritics & Planetary Science, 44(9), 1243-1264 (2009).

[13] Matsumoto, T., Tsuchiyama, A., Miyake, A., Noguchi, T., Nakamura, M., Uesugi, K., Takeuchi, A., Suzuki, Y. & Nakano, T. Surface and internal structures of a space-weathered rim of an Itokawa regolith particle. Icarus, 257, 230-238 (2015).

[14] Laczniak, D.L., Thompson, M.S., Christoffersen, R., Dukes, C.A., Clemett, S.J., Morris, R.V. & Keller, L.P. Characterizing the spectral, microstructural, and chemical effects of solar wind irradiation on the Murchison carbonaceous chondrite through coordinated analyses. Icarus, 364, p.114479. (2021).

[15] Laczniak, D.L., Thompson, M.S., Christoffersen, R., Dukes, C.A., Morris, R.V. & Keller, L.P. Investigating the role of incident ion flux in solar wind space weathering of carbon-rich asteroidal regolith via H+ and He+ irradiation of the Murchison meteorite. Icarus, 410, p.115883 (2024).

[16] Daly, R. T., Bierhaus, E. B., Barnouin, O. S., Daly, M. G., Seabrook, J. A., Roberts, J. H., Ernst, C. M., Perry, M. E., Nair, H., Espiritu, R. C., Palmer, E. E., Gaskell, R. W., Weirich, J. R., Susorney, H. C. M., Johnson, C. L., Walsh, K. J., Nolan, M. C., Jawin, E. R., Michel, P., Trang, D. & Lauretta, D. S. The morphometry of impact craters on Bennu. Geophysical Research Letters, 47, e89672 (2020).

[17] Lauretta, D. S., Adam, C. D., Allen, A. J., Ballouz, R.-L., Barnouin, O. S., Becker, K. J., Becker, T., Bennett, C. A., Bierhaus, E. B., Bos, B. J., Burns, R. D., Campins, H., Cho, Y., Christensen, P. R., Church, E. C. A., Clark, B. E., Connolly Jr., H. C., Daly, M. G., DellaGiustina, D. N., Drouet d'Aubigny, C. Y., Emery, J. P., Enos, H. L., Freund Kasper, S., Garvin, J. B., Getzandanner, K., Golish, D. R., Hamilton, V. E., Hergenrother, C. W., Kaplan, H. H., Keller, L. P., Lessac-Chenen, E. J., Liounis, A. J., Ma, H., McCarthy, L. K., Miller, B. D., Moreau, M. C., Morota, T., Nelson,D. S., Nolau, J. O., Olds, R., Pajola, M., Pelgrift, J. Y., Polit, A. T., Ravine, M. A., Reuter, D. C., Rizk, B., Rozitis, B., Ryan, A. J., Sahr, E. M., Sakatani, N., Seabrook, J. A., Selznick, S. H., Skeen, M. A., Simon, A. A., Sugita, S., Walsh, K. J., Westermann, M. M., Wolner, C. W. V. & Yumoto, K. Spacecraft sample collections and subsurface excavation of asteroid (101955) Bennu. Science 377, 285-291. doi/10.1126/science.abm1018 (2022).

[18] Noguchi, T., Matsumoto, T., Miyake, A., Igami, Y., Haruta, M., Saito, H., Hata, S., Seto, Y., Miyahara, M., Tomioka, N. & Ishii, H.A. A dehydrated space-weathered skin cloaking the hydrated interior of Ryugu. Nature Astronomy, 7, 170-181 (2023).

[19] Matsuoka, M., Kagawa, E., Amano, K., Nakamura, T., Tatsumi, E., Osawa, T., Hiroi, T., Milliken, R., Domingue, D., Takir, D., Brunetto, R., Barucci, A., Kitazato, K., Sugita, S., Fujioka, Y., Sasaki, O., Kobayashi, S., Iwata, T., Morota, T., Yokota, Y., Kouyama, T., Honda, R., Kameda, S., Cho, Y., Yoshioka, K., Sawada, H., Hayakawa, M., Sakatani, N., Yamada, M., Suzuki, Hidehiko H., Chikatoshi O., K., Shirai, K., Lantz, C., R., Stefano Y., H., Noguchi, T., Okazaki, R., Yabuta, H., Naraoka, H., Sakamoto, K., Tachibana, S., Yada, T., Nishimura, M., Nakato, A., Miyazaki, A., Yogata, K., Abe, M., Okada, T.,, Usi, T., Y., Makoto S., Takanao T., S.,, F., Nakazawa, S., Watanabe, S., and Tsuda, Y. (2023) Space weathering acts strongly on the uppermost surface of Ryugu. Communications: Earth & Environment, 4, 335 (2023). https://doi.org/10.1038/s43247-023-00991-3.

[20] Lauretta, D. S., H. C. Connolly Jr., J. E. Aebersold, C. M. O. D. Alexander, R-L. Ballouz, J. J. Barnes, H. C. Bates, C. A. Bennett, L. Blanche, E. H. Blumenfeld, S. J. Clemett, G. D. Cody, D. N. DellaGiustina, J. P. Dworkin, S. Eckley, D. I. Foustoukos, I. A. Franchi, D. P. Glavin, R. C. Greenwood, P. Haenecour, V. E. Hamilton, D. H. Hill, T. Hiroi, K. Ishimaru, F. Jourdan, H. H. Kaplan, L. Keller, A. J. King, P/ Koefoed, M. K. Kontogiannis, L. Le, R. J. Macke, T. J. McCoy, R. E. Milliken, J. Najorka, A. N. Nguyen, M. Pajola, A. T. Polit, H. L. Roper, S. S. Russell, A. J. Ryan, S. A. Sandford, P. F. Schofield, C. D. Schultz, L. B. Seifert, S. Tachibana, K. L. Thomas-Keprta, M. S. Thompson, V. Tu, F. Tusberti, K. Wang, T. J. Zega, C. W. V. Wolner & OSIRIS-REx Sample Analysis Team. Asteroid (101955) Bennu in the laboratory: Properties of the sample collected by OSIRIS-REx, Meteoritics and Planetary Science, DOI: 10.1111/maps.14227 (2024).

[21] Hamilton, V. E. Simon, A. A., Christensen, P. R., Reuter, D. C., Clark, B. E., Barucci, M. A., Bowles, N. E., Boynton, W. V., Brucato, J. R., Cloutis, E. A., Connolly, H. C., Donaldson Hanna, K. L., Emery, J. P., Enos, H. L., Fornasier, S., Haberle, C. W., Hanna, R. D., Howell, E. S., Kaplan, H. H., Keller, L. P., Lantz, C., Li, J.-Y., Lim, L. F., McCoy, T. J., Merlin, F., Nolan, M. C., Praet, A., Rozitis, B., Sandford, S. A., Schrader, D. L., Thomas, C. A., Zou, X.-D., Lauretta, D. S. & OSIRIS-REx Team. Evidence for widespread hydrated minerals on asteroid (101955) Bennu. Nature Astronomy, doi: 10.1038/s41550-019-0722-2 (2019).

[22] Liu, Y., Guan, Y., Zhang, Y., Rossman, G.R., Eiler, J.M. and Taylor, L.A. Direct measurement of hydroxyl in the lunar regolith and the origin of lunar surface water. Nature Geoscience, 5, 779-782 (2012).

[23] Zhou, C., Tang, H., Li, X., Zeng, X., Mo, B., Yu, W., Wu, Y., Zeng, X., Liu, J. and Wen, Y. Chang'E-5 samples reveal high water content in lunar minerals. Nature Communications, 13, 5336 (2022).

Table S1: DOIs of the instrument data products underlying the figures and tables.

Figure or Table	DOI
Figure 1	10.60707/pcey-ga67
Figure 2a, b	10.60707/dkyw-4n60
Figure 2c	10.60707/86p0-7h18
Figure 2d	10.60707/ssys-1p19
Figure 2e	10.60707/t41a-sk39
Figure 3	10.60707/frgz-tt08
Figure 4 a,c,d	10.60707/7mnt-tx36; 10.60707/av9y-vs03
Figure 4 b,e,f	10.60707/jm4a-rp49; 10.60707/ae1y-7b21
Figure 4g	10.60707/002g-5h90; 10.60707/pte6-nf07
Figure 5a	10.60707/jyc8-t984
Figure 5b,c	10.60707/85mp-k210
Figure 6a, b	10.60707/4105-wk41
Figure 6c,d	10.60707/3901-pk48
Table S3	10.60707/j6f9-tp89
Table S2	10.60707/cx75-4h42
Table S2	10.60707/81r9-tv08
Table S2	10.60707/1dst-en63
Table S2	10.60707/rm08-bg04
Figure ED1	10.60707/k5d7-jv15
Figure ED2a,b,c	10.60707/881y-gp93; 10.60707/jyzz-pt45; 10.60707/7mpt-a743; 10.60707/h62h-tz95; 10.60707/t05d-ff37
Figure ED2d	10.60707/jm4a-rp49
Figure ED2e,f	10.60707/nca7-7876

Table S2. Melt deposit compositions. Energy-dispersive X-ray analyses (in at. %) of melt deposit bulk compositions for samples OREX-501017-100 (columns 1 and 2) and OREX-501017-101 (columns 3 and 4).

	1	2	3	4
O	59.07	59.2	58.12	58.08
Mg	13.05	12.37	13.14	13.17
Al	1.03	0.92	0.91	0.93
Si	12.74	12.69	11.66	11.64
S	2.41	2.57	2.01	1.99
Ca	0.25	0.24	0.6	0.69
Cr	0.18	0.18	0.18	0.15
Mn	0.11	0.13	0.13	0.15
Fe	10.83	11.26	12.88	12.84
Ni	0.33	0.44	0.37	0.36
Total	100	100	100	100

Table S3. Sample compositions for radionuclide studies. Measured concentrations of major elements, Mg, Al, S, Ca, Mn, Fe, and Ni (in wt.%), minor elements K, Ti, Co (in ppm) and the cosmogenic radionuclides ^{10}Be, ^{26}Al and ^{36}Cl (in dpm/kg) in OREX-803047-0, a 9.85 mg split of OREX-803014-0.

Element/Nuclide	Concentration
Mg (wt.%)	10.5
Al (wt.%)	0.90
S (wt.%)	6.6
K (ppm)	540
Ca (wt.%)	1.03
Ti (ppm)	470
Mn (ppm)	0.20
Fe (wt.%)	20.1
Co (ppm)	560
Ni (wt.%)	1.18
10Be	7.80 ± 0.17
26Al	21.1 ± 0.7
36Cl	91.3 ± 1.7
